# GLP‐1 Receptor Agonists in Brazil: Landscape of Consumption, Safety and Regulation

**DOI:** 10.1111/dom.70609

**Published:** 2026-03-08

**Authors:** Renato Lopes Hurtado, Angélica Amorim Amato, Hugo de Luca Corrêa, Dâmaris Silveira

**Affiliations:** ^1^ Agência Nacional de Vigilância Sanitária (Anvisa) Brasília Brazil; ^2^ University of Brasília Brasília Brazil; ^3^ Catholic University of Brasília Brasília Brazil

**Keywords:** GLP‐1 analogue, pharmaco‐epidemiology, population study, real‐world evidence

## Abstract

**Background:**

The escalating use of Glucagon‐like peptide‐1 receptor agonists (GLP‐1 RAs) is reshaping pharmaceutical consumption patterns and posing unprecedented challenges to healthcare systems worldwide. This study analyzes the landscape of GLP‐1 RA consumption, safety monitoring and regulatory oversight in Brazil.

**Methods:**

A retrospective regulatory surveillance study was carried out triangulating data from three official sources: (1) national sales covering 2020–2024, classifying according to Anatomical Therapeutic Chemical (ATC) system; (2) adverse event reports submitted to Vigimed, the national pharmacovigilance platform integrated with global VigiBase; and (3) official alerts of falsified products. Sales data were sourced from industrial reports and the National System for the Management of Controlled Products (SNGPC).

**Results:**

Semaglutide emerged as the predominant GLP‐1 RA in the Brazilian market. Consumption was markedly concentrated in regions with higher Gross Domestic Product (GDP), revealing a disconnect between medication use and regional diabetes prevalence. Pharmacovigilance analysis uncovered a significant proportion of reports involving off‐label use, pointing to potential clinical and regulatory gaps. Furthermore, documented cases of counterfeit products underscore critical supply chain vulnerabilities.

**Conclusion:**

The convergence of escalating demand, widespread off‐label use and product falsification requires a coordinated and agile regulatory response. The observed trends suggest significant access disparities driven by economic factors rather than epidemiological need.

## 1 Introduction

The global burden of obesity and type 2 diabetes (T2D) presents a critical challenge to health systems. Glucagon‐like peptide‐1 receptor agonists (GLP‐1 RAs) have emerged as a cornerstone therapy due to their well‐established efficacy in glycemic control and significant weight loss outcomes reported in major clinical trials [1, 2]. Evidence shows that semaglutide and tirzepatide reduce heart failure hospitalisations or all‐cause mortality by over 40% in patients with cardiometabolic heart failure [3]. Tirzepatide also produces substantial and sustained weight loss, with a 25.3% reduction over 88 weeks versus 9.9% with placebo, whereas treatment interruption leads to marked weight regain [4]. Consequently, the demand for these agents has surged worldwide, reshaping the pharmaceutical landscape.

Although the clinical benefits of GLP‐1 RAs are widely recognised, the real‐world implications of their rapid diffusion in developing pharmaceutical markets remain underexplored. In Brazil, this phenomenon is particularly complex. The widespread popularity of these drugs, often fueled by social media trends and off‐label use for cosmetic weight loss, has outpaced traditional surveillance mechanisms [5]. Emerging data also point to rare but serious complications, including pancreatitis, gastroparesis and intestinal obstruction, highlighting the need for mitigation strategies [6]. This scenario raises urgent questions regarding equitable access, patient safety and the integrity of the supply chain.

This study aims to characterise the landscape of GLP‐1 RA consumption in Brazil from a regulatory surveillance perspective. Unlike purely clinical studies, we triangulate data from official national sales reports, pharmacovigilance notifications, and falsification alerts. Specifically, we examine consumption trends, the profile of adverse event reporting, and the emergence of a counterfeit market, providing a comprehensive overview of the challenges faced by the Brazilian health system [7].

## 2 Materials and Methods

A mixed‐methods, document‐based observational design was employed, combining a quantitative descriptive component with a qualitative interpretative component to characterise utilisation, safety signals, falsification events and regulatory responses related to GLP‐1 receptor agonists (GLP‐1 RAs) in Brazil. Data were obtained from official information systems maintained by the Brazilian Health Regulatory Agency (Anvisa) and from publicly available regulatory documents. Because these systems are independent and non‐integrated, record‐level linkage was not feasible; analyses were therefore descriptive and interpreted as regulatory surveillance indicators rather than individual exposure–outcome estimates.

Observation periods were defined a priori to align with system implementation milestones, regulatory enforcement changes and data availability, thereby maximising temporal comparability and reducing measurement bias across sources. National sales volumes (2020–2024) were extracted from Anvisa's Statistical Yearbook of the Pharmaceutical Market, available at Anvisa's website [8]. Although the National System for the Management of Controlled Products (SNGPC) is the official electronic platform for transmitting dispensing data for medicines subject to prescription retention, mandatory and strictly enforced recording of GLP‐1 RAs was implemented only in 2025; therefore, historical trend analyses for 2020–2024 relied on aggregated industrial supply/sales reports to avoid under‐registration biases that could affect earlier SNGPC data. Dispensing analyses using SNGPC were conducted from June 2025 onward, after mandatory reporting for the drug class of interest became operational.

Pharmacovigilance data were extracted from VigiMed, Anvisa's national reporting system integrated with the WHO global database (VigiBase). The analysis window (January 2024 to March 2025) was selected to reflect the period when the system and relevant coding practices were fully operational for the taxonomies analyzed, thereby supporting improved consistency and international comparability after migration from legacy systems. Confirmed falsification events were identified from Anvisa's official alerts (January 2024–September 2025). A falsification episode was classified as confirmed only when it met the standard regulatory confirmation pathway: (i) suspect products are identified via complaints; (ii) technically assessed by the marketing authorisation holder (manufacturer); formally confirmed before the issuance of an official alert.

Utilisation was described as package/unit volumes and as population‐standardised rates. Where presentation‐level strength and pack content were available (national sales data), utilisation was additionally expressed as WHO Defined Daily Doses (DDDs) using the ATC/DDD methodology (Appendix S1). DDDs were used solely as a standardisation metric to improve comparability across molecules and presentations; however, DDDs do not necessarily reflect prescribed or actual doses and may differ substantially from real‐world dosing for GLP‐1 RAs, particularly when used for obesity treatment and during titration or maintenance phases. In contrast, DDD‐based utilisation could not be computed consistently for SNGPC dispensing exports due to incomplete or non‐standardised mapping between product presentations, strengths and pack content; therefore, SNGPC results are presented as package volumes and, when applicable, population‐standardised package rates, and should be interpreted as an administrative proxy rather than a precise pharmacoepidemiologic exposure measure.

Prescriber categories in SNGPC were grouped as physicians, dentists and veterinarians. Entries labelled ‘RMS’ (Registro do Ministério da Saúde) were classified as ‘Other’, because the system does not identify the specific professional category for these records.

To explore socioeconomic and epidemiological correlates of GLP‐1 RA use, we performed state‐level ecological analyses using non‐parametric Spearman correlations based on population‐standardised dispensing rates. The primary outcome was the number of dispensed units per 100 000 inhabitants (industrial products), calculated by dividing the total dispensed units in each state by its resident population and multiplying by 100 000. Population denominators were obtained from official Brazilian Institute of Geography and Statistics (IBGE) population estimates published in the Diário Oficial da União (reference date: July 1, 2024) [9]. Explanatory variables included Gross Domestic Product (GDP) per capita (IBGE, 2022) [10] and adult obesity prevalence (%) from Vigitel 2023 (Ministry of Health) [11]. Because Vigitel estimates are based on residents of the 26 state capitals and the Federal District and rely on self‐reported anthropometric data, obesity prevalence was treated as an imperfect proxy for statewide prevalence, with potential systematic measurement error that could attenuate or distort correlation magnitudes. Accordingly, ecological results were interpreted as hypothesis‐generating and not as evidence of causality or individual‐level association. Variables were organised in a state × indicator matrix and evaluated using Spearman's rank correlation coefficient (*ρ*), with *α* = 0.05. Analyses were conducted in Python 3.12.x using pandas and scipy.stats.

### 2.1 Use of AI‐Assisted Writing

ChatGPT was used to refine the clarity, organisation and formatting of the manuscript text. AI tools were not used for data extraction, statistical analysis, result generation or interpretation. All scientific content was reviewed, edited and remains the full responsibility of the authors.

### 2.2 Ethics

The project was approved by the Research Ethics Committee of the Faculty of Medicine, University of Brasília (CEP/FM‐UnB), Decision Report No. 7941474 (November 1, 2025), with exemption from CONEP because it is an observational, retrospective study using only secondary data, in accordance with CNS Resolution 466/12.

## 3 Results and Discussion

### 3.1 Consumption of GLP‐1 Receptor Agonists

**FIGURE 1:**
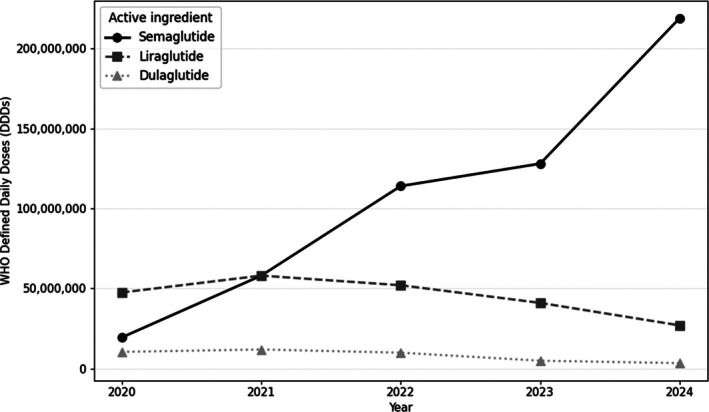
Annual national utilisation of GLP‐1 receptor agonists in Brazil (2020–2024), expressed as total WHO Defined Daily Doses (DDDs).

Using national sales data (2020–2024), we observed a sustained expansion in the utilisation of GLP‐1 receptor agonists (GLP‐1 RAs) in Brazil, expressed both as packages and as WHO‐defined daily doses (DDDs). This growth was largely driven by semaglutide, whereas liraglutide and dulaglutide showed stable‐to‐declining trends over the same period. Because DDDs reduce the influence of presentation and pack‐size heterogeneity, Figure 1 summarises national utilisation as total DDDs to facilitate cross‐molecule comparability. Notably, the steepest year‐over‐year increase in DDDs is observed after 2023. Tirzepatide is not included in the 2020–2024 series because it was commercialised in Brazil after 2025.

To strengthen the interpretation of these trajectories, we contextualised the sales series using the regulatory incorporation timeline. This post‐2023 inflexion is temporally aligned with regulatory milestones, including Anvisa's approval of semaglutide for obesity/overweight in January 2023. Table 1 summarises Anvisa authorisation milestones and therapeutic indications for GLP‐1 RAs in Brazil. These milestones provide a regulatory framework for interpreting changes in national utilisation patterns over time; however, temporal alignment should not be interpreted as causal attribution, since aggregate sales data do not capture indication‐specific prescribing (e.g., diabetes vs. obesity), patient‐level switching or clinical decision pathways.

**TABLE 1:** Authorisation of GLP‐1 analogues in Brazil and their therapeutic indications.

Active ingredient	Indication	Approval date
Liraglutide	Type 2 diabetes	March 29, 2010
Dulaglutide	Type 2 diabetes	August 31, 2015
Liraglutide	Obesity/overweight	February 29, 2016
Semaglutide	Type 2 diabetes	August 6, 2018
Semaglutide	Obesity/overweight	January 2, 2023
Tirzepatide	Type 2 diabetes	September 25, 2023

*Note*: Anvisa authorisation timeline for GLP‐1 receptor agonists in Brazil and corresponding therapeutic indications. These milestones provide the regulatory context for interpreting inflexion points observed in the national utilisation series (Figure 1).

Although the sales data clearly document a shift towards newer agents, statements regarding therapeutic substitution, patient preferences and evolution of clinical practice cannot be inferred directly from sales volumes alone. They should be framed as contextual explanations supported by external evidence. International clinical trials have shown that newer incretin‐based agents achieve greater mean weight loss than earlier options, plausibly increasing demand once products become available and indications expand. For example, once‐weekly semaglutide 2.4 mg produced clinically meaningful weight reduction in adults with overweight/obesity, and tirzepatide produced substantial weight loss in adults with obesity, reinforcing why new‐generation incretin therapies have reshaped obesity pharmacotherapy globally [12, 13]. In addition, real‐world studies have reported higher persistence and adherence for once‐weekly semaglutide compared with other GLP‐1 RAs, and consistent differences in adherence/persistence across GLP‐1 RA regimens in nationwide routine‐care data [14, 15]. Taken together, the Brazilian sales patterns observed here are consistent with a broader international shift towards newer incretin therapies; however, the drivers of this shift should be interpreted as plausible contextual factors grounded in the literature rather than as direct inferences from sales data.

### 3.2 Counterfeit Cases Involving GLP‐1 Receptor Agonists

Between January 2024 and September 2025, eight confirmed episodes of falsification were identified, involving seven cases of semaglutide and one case of liraglutide. In none of the eight cases was it possible to identify the responsible parties, and no reports of adverse health effects associated with these occurrences have been received to date.

Importantly, the number of confirmed episodes should not be interpreted as evidence that falsification is a marginal issue. Our dataset captures only cases that progress through a conservative confirmation pathway: initial suspicion or complaint, technical assessment and formal confirmation by the marketing authorisation holder, and subsequent publication of an official Anvisa alert, so it likely underestimates the true volume of falsified products in circulation. Consequently, these eight confirmed episodes likely represent the ‘tip of the iceberg’, as additional suspect products may circulate without detection. This under‐detection limitation strengthens the public health interpretation: even a small number of confirmed episodes signals vulnerabilities in traceability and supply‐chain integrity. It supports the need for sustained post‐marketing surveillance, risk communication and enforcement actions to reduce exposure to falsified medicines.

### 3.3 Pharmacovigilance

**FIGURE 2:**
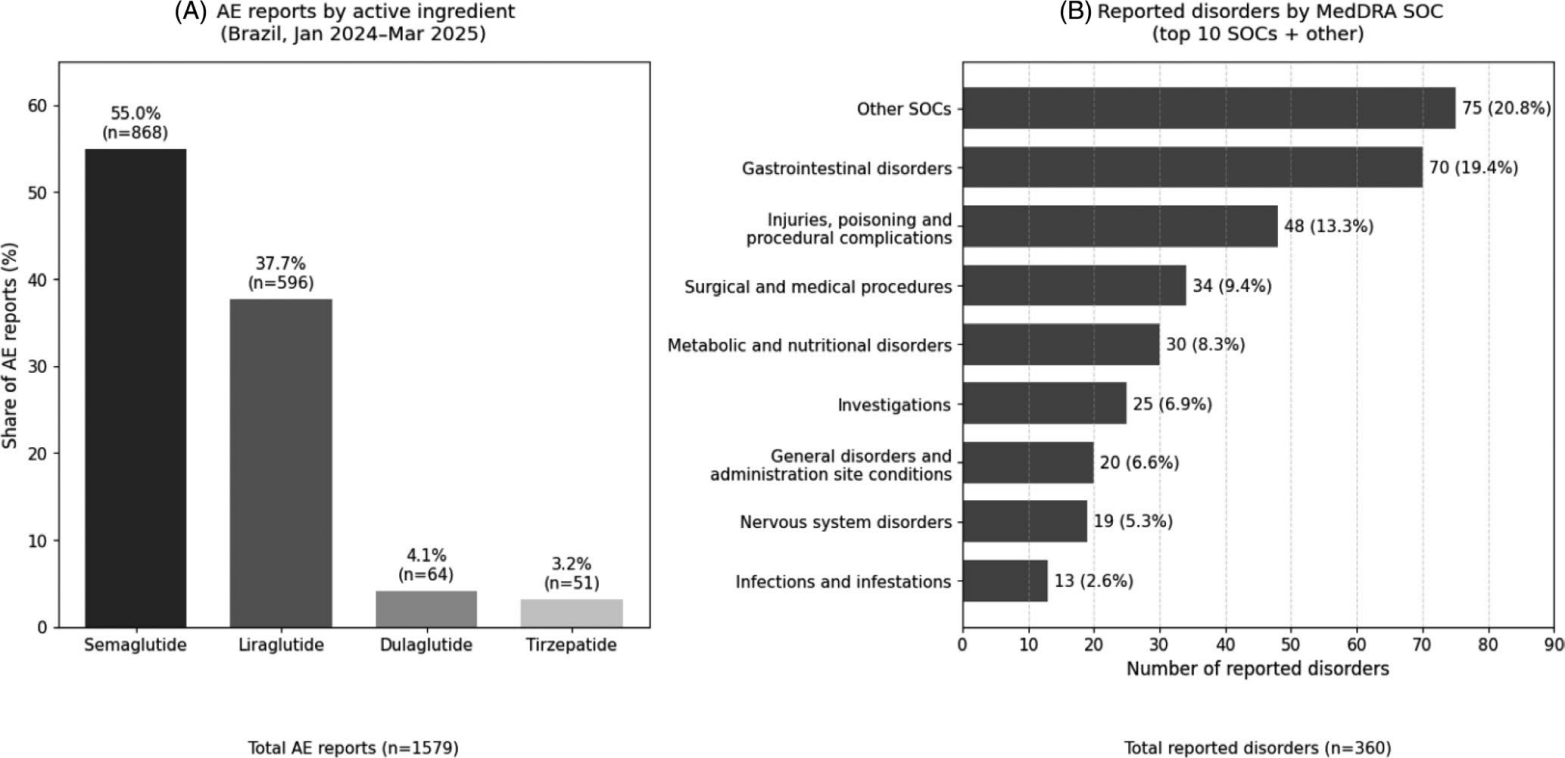
Combined pharmacovigilance summary for GLP‐1 receptor agonists in Brazil (January 2024–March 2025). (A) Share of spontaneous adverse event (AE) reports by active ingredient (total AE reports: *n* = 1579). (B) Distribution of reported disorders by MedDRA System Organ Class (SOC), showing the top 10 SOCs plus aggregated ‘Other SOCs’ (total reported disorders: *n* = 360). Panel A reflects the composition of spontaneous reports (not incidence or risk). Panel B reflects the number of reported disorder entries categorised by SOC and therefore uses a different denominator.

Regarding adverse events (AEs), 1579 notifications were recorded in Brazil between Jan 2024 and Mar 2025 (868 involving semaglutide, 596 involving liraglutide, 64 involving dulaglutide, and 51 involving tirzepatide). As shown in Figure 2, semaglutide alone contributed 55.0% of all GLP‐1 RA AE reports, and together with liraglutide accounted for 92.8% of notifications. Of these reports, 94.5% involved registered products, and most notifications originated from the marketing authorisation holders. VigiMed data further indicated that semaglutide‐related AEs explicitly associated with off‐label use were proportionally higher in Brazil than in the WHO global database. Globally, off‐label use accounts for approximately 7.8% of semaglutide AE reports, whereas in Brazil, it accounts for 38% of the 886 semaglutide notifications. Importantly, these proportions refer to the composition of spontaneous reports and should not be interpreted as incidence, comparative risk or population‐level frequency, given underreporting, reporting biases and the absence of an exposure denominator [16]. Because the underlying information systems are independent, these data cannot be extrapolated to the overall utilisation recorded in SNGPC, nor can they be directly linked to falsification cases.

Gastrointestinal and metabolic manifestations predominated among AEs (mostly mild to moderate), with less frequent but clinically significant severe outcomes.

Building on the SOC distribution shown in Figure 2, pancreatitis‐related terms accounted for a notable share of Brazilian spontaneous reports (5.9% of notifications), more than double the proportion reported in the WHO global database (2.4%) [16]. Figure 2A,B describes the distribution of reported terms within spontaneous notifications and should be interpreted in the context of underreporting, variable reporting quality and reporting biases. Moreover, an elevated proportion of pancreatitis‐related terms may reflect stimulated reporting, heightened clinical awareness and differential ascertainment (including increased attention during periods of rapid diffusion and high public visibility), rather than a true increase in clinical incidence. Accordingly, this pattern should be viewed primarily as a reporting signal warranting continued monitoring and contextual evaluation, rather than as evidence of higher pancreatitis risk in Brazil.

Table 2 presents the number of dispensing cases of GLP‐1 analogues in Brazil, based on prescriptions issued via the SNGPC system, distinguishing between compounded and industrialised products since June 2025.

**TABLE 2:** Dispensing of GLP‐1 analogues in Brazil based on SNGPC prescriptions (June 2025 onward).

Substance	Origin	Units (dispensed)	Physicians	Dentists	Veterinarians	Others
Semaglutide	Industrialised	318 331	316 516	1542	204	69
	Compounded	3216	3154	62	0	0
Tirzepatide	Industrialised	181 677	179 992	1488	163	34
	Compounded	3595	3125	451	17	2
Liraglutide	Industrialised	64 776	63 882	794	81	19
	Compounded	NI*	NI	NI	NI	NI
Dulaglutide	Industrialised	6408	6273	114	14	7
	Compounded	NI*	NI	NI	NI	NI
Total	—	577 923	572 942	4451	476	134

*Note*: SNGPC dispensing volumes are administrative records and do not represent exposure duration, adherence or clinical outcomes.

Abbreviation: NI, Not Informed.

SNGPC data also included prescriptions recorded as originating from dentists and veterinarians. Interpreting this finding requires distinguishing between legal permissibility and prescribing authority, as defined by national legislation, professional regulation and scope‐of‐practice norms established by professional councils, and patient safety implications. There is no single specific law in Brazil that explicitly defines which professional categories are authorised to prescribe this class of medicines; oversight of prescribing practices is primarily exercised by each professional council within its regulatory mandate. From a surveillance perspective, these records signal potentially non‐standard prescribing pathways and underscore the importance of risk communication, rational‐use guidance and professional oversight to ensure prescribing aligns with evidence‐based indications, adequate monitoring and patient safety considerations.

In parallel, the rapid diffusion of GLP‐1 analogues increases the importance of structured initiation protocols, longitudinal follow‐up and clear communication to reduce preventable medication‐use problems. Documented cases of accidental semaglutide overdose illustrate the relevance of standardised initiation and educational strategies [17]. More broadly, clinical governance and structured care pathways may improve safe‐use practices in settings of high demand [18]. Evidence also suggests that multiprofessional follow‐up, particularly when pharmaceutical counselling is available, can support adherence, reduce errors, and facilitate earlier recognition and management of adverse reactions [19, 20, 21].

According to Ivama‐Brummell et al. [22], 1165 AE reports were recorded in Brazil, with liraglutide (548) and semaglutide (542) accounting for 92% of occurrences. The temporal distribution showed distinct profiles: liraglutide showed accumulation over 13 years (median of 7 per year), whereas semaglutide showed a concentration within its 6 years of commercialisation (median of 110 per year). Thirty‐eight percent of semaglutide reports corresponded to off‐label use. For liraglutide, off‐label reporting remained low; for dulaglutide, none were reported; and for tirzepatide, 11 off‐label notifications were reported.

The adverse event profile demonstrated a predominance among women of reproductive age, with statistically significant signals for semaglutide and tirzepatide, including 23.4% of notifications involving off‐label use [22]. In this study, the most frequently reported adverse events, predominantly mild to moderate gastrointestinal symptoms, were consistent with the published literature [23, 24]. Overall, these findings support continued pharmacovigilance, targeted risk communication, and evidence‐based guidance to prescribers and users as utilisation expands.

Semaglutide generated the largest volume of notifications within a shorter period, consistent with its rapid market growth and increased visibility after broader adoption. Liraglutide displayed a more dispersed temporal pattern, reflecting a longer commercialisation history. These differences likely reflect both the stage of incorporation of each molecule and reporting dynamics in contexts of high demand and public attention. Public health campaigns and professional guidance should emphasise multidisciplinary care to complement GLP‐1 therapy, optimise outcomes, and address broader determinants of obesity and metabolic health [25].

### 3.4 Regulatory Response in Brazil

In recent years, Anvisa has implemented a structured set of regulatory actions to mitigate the increasing risks associated with the widespread use of GLP‐1 receptor agonists. The first action, issued in 2024, focused on perioperative safety, alerting healthcare professionals to the risk of delayed gastric emptying during procedures requiring anaesthesia or deep sedation and recommending specific precautions [26].

In 2025, Anvisa adopted additional measures to reinforce regulatory oversight. One set of actions introduced mandatory prescription retention, limited validity of medical prescriptions, and compulsory SNGPC reporting for GLP‐1 receptor agonists. These requirements, implemented progressively across the country, aimed to strengthen traceability and improve regulatory control of prescribing and dispensing practices [27, 28].

Another regulatory action addressed the compounding and importation of components used in pen injectors for these medicines. By establishing clearer rules and quality standards, Anvisa sought to enhance surveillance over the supply chain and reduce the risk of unsafe or irregular products entering the market [29].

As compounded GLP‐1 APIs gained visibility, the Agency issued technical guidance to harmonise regulatory interpretation regarding their use. The guidance reaffirmed that APIs may only be compounded when an approved industrialised medicine containing the same substance exists. It also clarified that biotechnological APIs, such as semaglutide, may only be compounded if sourced from the same manufacturer evaluated in the original approval. In contrast, synthetic APIs, such as tirzepatide, may be compounded with appropriate quality‐control testing.

Collectively, these coordinated actions reflect the regulatory intent to strengthen safety, traceability, and public health protection amid rapid market expansion. However, regulatory intent should be distinguished from demonstrated outcomes. Assessing whether these measures reduce off‐label use, curb falsification, or measurably change dispensing patterns is beyond the scope of the current study. It will require longitudinal follow‐up using designs capable of evaluating changes over time.

Taken together, these regulatory measures underscore that observed utilisation patterns should be interpreted not only in terms of overall growth, but also in terms of how access and structural conditions shape geographic variation in use.

### 3.5 Determinants of Consumption

The findings indicate a more nuanced consumption pattern than a simple ‘need‐driven’ dynamic. As shown in Table 3, no significant correlation was observed between per capita GLP‐1 RA consumption and obesity prevalence across Brazilian states (Spearman's *ρ* = −0.16; *p* = 0.415), suggesting that states with a higher obesity burden did not necessarily exhibit greater GLP‐1 agonist use when adjusted for population size. In this context, the geographic distribution of use appears to reflect broader structural and contextual factors—such as socioeconomic conditions, health system organisation, and access to specialised care—indicating that consumption is shaped not only by epidemiological need but also by unequal access and other social determinants.

**TABLE 3:** Population‐standardised dispensing of industrialised GLP‐1 RAs by Brazilian state (SNGPC report), with obesity prevalence (Vigitel 2023) and GDP per capita (IBGE 2022).

UF	Dispensed units (*n*)	Population (IBGE 2024)	Units per 100 000	Obesity (%) capitals 2023	GDP per capita 2022 (BRL)
Acre	195	880 631	22.1	25.3	28 525
Alagoas	5961	3 220 104	185.1	22.0	24 322
Amazonas	1528	4 281 209	35.7	25.6	36 827
Amapá	1453	802 837	181.0	30.4	32 194
Bahia	20 614	14 850 513	138.8	25.8	28 483
Ceará	20 164	9 233 656	218.4	27.8	24 296
Distrito Federal	16 815	2 982 818	563.7	19.3	116 713
Espírito Santo	10 646	4 102 129	259.5	19.4	47 619
Goiás	22 779	7 350 483	309.9	17.7	45 156
Maranhão	8062	7 010 960	115.0	20.9	20 633
Minas Gerais	64 804	21 322 691	303.9	21.3	44 147
Mato Grosso do Sul	8888	2 901 895	306.3	27.8	60 365
Mato Grosso	5222	3 836 399	136.1	26.7	69 839
Pará	12 490	8 664 306	144.2	25.3	29 095
Paraíba	3916	4 145 040	94.5	22.5	21 662
Pernambuco	18 703	9 539 029	196.1	26.3	27 139
Piauí	5581	3 375 646	165.3	20.6	22 279
Paraná	30 242	11 824 665	255.8	23.3	53 710
Rio de Janeiro	55 918	17 219 679	324.7	26.6	71 850
Rio Grande do Norte	5253	3 446 071	152.4	22.4	28 409
Rondônia	2688	1 746 227	153.9	23.4	33 153
Roraima	869	716 793	121.2	21.4	42 248
Rio Grande do Sul	16 596	11 229 915	147.8	28.1	54 559
Santa Catarina	16 286	8 058 441	202.1	21.3	61 274
Sergipe	6347	2 291 077	277.0	25.2	25 965
São Paulo	278 487	45 973 194	605.8	24.6	70 471
Tocantins	2648	1 577 342	167.9	20.6	38 512

*Note*: Spearman correlations (state level): units per 100 000 vs. obesity (*ρ* = −0.16, *p* = 0.415); units per 100 000 vs. GDP per capita (*ρ* = 0.50, *p* = 0.008).

Moreover, Table 3 shows that GLP‐1 RA consumption was significantly associated with state‐level economic capacity (Spearman *ρ* = 0.50; *p* = 0.008). Taken together, these results suggest that access to these medicines is strongly modulated by regional wealth, reinforcing the interpretation that consumption patterns follow gradients of economic capacity rather than solely the distribution of clinical need.

In this context, national chronic disease surveillance provides relevant background. Vigitel 2023 reports substantial burdens of obesity and diabetes in Brazilian capitals, with heterogeneity across regions; however, these estimates are restricted to capital cities and rely on self‐reported data, limiting their use as a direct proxy for statewide epidemiological burden. Accordingly, the absence of correlation with obesity and the positive association with GDP per capita are compatible with an access gradient shaped by socioeconomic and health‐system factors rather than reflecting the distribution of need alone.

These disparities are also unlikely to be explained solely by formal differences in regulated price ceilings across states. In Brazil, maximum consumer prices are regulated at the national level by the Drug Market Regulation Chamber (CMED) through ceiling prices (PMC), which constrains list‐price variation [30]. Therefore, the observed association with GDP per capita is compatible with inequality in affordability and access, rather than with differences in regulated maximum prices.

Against this backdrop, CONITEC's (National Committee for Health Technology Incorporation) recommendation not to incorporate liraglutide and semaglutide into the Brazilian public health system (SUS) for obesity treatment becomes particularly relevant. By keeping these therapies outside public coverage, this policy, although grounded in cost‐effectiveness assessment, sustains an income‐based barrier to access and, consequently, perpetuates socioeconomic inequalities in treatment availability.

### 3.6 Future Directions and Technology Transfer

In parallel, following CONITEC's recommendation against incorporating GLP‐1 RAs into the SUS, the Ministry of Health has been negotiating strategies to enable a more equitable future incorporation through domestic production and technology transfer. Notable initiatives include public‐private partnerships for local manufacturing of GLP‐1 RAs, structured in phases (initially filling/finishing with imported API, followed by domestic API production). This strategy aims to create the budgetary conditions needed to expand access, thereby laying the groundwork for future incorporation of GLP‐1 analogues into the SUS [31].

## 4 Study Limitations

This study has limitations inherent to a regulatory surveillance design triangulating multiple administrative and document‐based sources. Because national systems are independent and non‐integrated, record‐level linkage across utilisation, pharmacovigilance, falsification alerts and regulatory actions was not possible; therefore, sales/dispensing patterns cannot be connected to specific adverse‐event reports, off‐label records, or falsification episodes. Findings should be interpreted as complementary surveillance indicators rather than person‐level exposure–outcome evidence. Data sources were also temporally misaligned: sales (2020–2024), Vigimed (January 2024–March 2025), falsification alerts (January 2024–September 2025), and SNGPC exports only after mandatory reporting (from June 2025), reflecting implementation milestones and availability, which limits cross‐system comparability and precludes robust evaluation of regulatory impact. Pharmacovigilance relies on spontaneous reporting and is subject to underreporting, differential and stimulated reporting, and incomplete information; thus, reported proportions (including off‐label and preferred terms) do not estimate incidence, event rates, comparative risk or causality. Utilisation metrics varied: DDDs were computed for sales where strength/pack data existed, but not for SNGPC due to missing standardised presentation/strength mapping; SNGPC results are presented as package volumes (and population‐standardised package rates) as an administrative proxy. Ecological analyses are hypothesis‐generating and constrained because obesity prevalence was based on Vigitel estimates, limited to state capitals, and on self‐reported anthropometry, introducing bias and limiting representativeness; the associations do not support individual inference or judgements about prescribing appropriateness. Overall, these constraints mean the study cannot inform individual‐level safety, effectiveness or prescribing decisions; instead, it serves as situational awareness and hypothesis generation rather than patient‐level risk/benefit estimates.

## 5 Conclusions

The rapid expansion of glucagon‐like peptide‐1 receptor agonist (GLP‐1 RA) use in Brazil represents a complex challenge that extends beyond clinical practice and requires sustained regulatory and public health attention. By triangulating complementary indicators from official sources, utilisation trends (sales and dispensing), spontaneous pharmacovigilance reports, and confirmed falsification alerts. This study provides a chronology of regulatory actions and an evidence‐informed situational overview intended for regulatory surveillance rather than a patient‐level pharmacoepidemiologic assessment.

Across data sources, the observed pattern is consistent with accelerated growth in use, driven by semaglutide, alongside a substantial off‐label footprint in spontaneous reports and recurring supply‐chain vulnerabilities reflected in confirmed falsification episodes. These findings should be interpreted as signals that support continued vigilance and targeted monitoring, not as estimates of incidence, comparative risk, or clinical effectiveness, given the absence of record‐level linkage, denominators and causal designs, as well as the known biases of spontaneous reporting systems.

Geographic variation in population‐standardised utilisation, and its closer alignment with economic capacity than with available capital‐based obesity prevalence indicators, likewise warrants cautious interpretation. These ecological patterns suggest, rather than confirm, structural and access‐related constraints that may shape availability and affordability. However, they do not provide definitive assessments of inequity or the appropriateness of prescribing at the individual level.

Taken together, these evidence‐informed signals reinforce the need for a coordinated response that integrates ongoing regulatory surveillance, strengthened pharmacovigilance capacity, risk communication to counter misinformation, and policy strategies to support safer and more equitable access as use continues to expand. Future work using longitudinal and evaluative designs will be essential to assess the impact of recent regulatory measures and to refine the governance of GLP‐1 RA use in Brazil.

## Funding

The authors have nothing to report.

## Conflicts of Interest

The authors declare no conflicts of interest.

## Supporting information


**Appendix S1:** Reference DDD values used (WHO ATC/DDD Index).

## Data Availability

Data openly available in a public repository as indicated at the references.
