# Supplementary material for: GLP‐1 Receptor Agonists in Brazil: Landscape of Consumption, Safety and Regulation
Source: Diabetes Obes Metab. 2026 Mar 8;28(5):4185–93. doi: 10.1111/dom.70609 (PMC13071213; doi:10.1111/dom.70609)
Supplement: Supplementary file 1 — Appendix S1: Reference DDD values used (WHO ATC/DDD Index). [file DOM-28-4185-s001.docx]

Appendix S1

## Reference DDD values used (WHO ATC/DDD Index)

| Drug | ATC code | DDD (mg) | Route | Source |
| --- | --- | --- | --- | --- |
| Liraglutide | A10BJ02 | 1.5 | P | WHO ATC/DDD Index (A10BJ02) |
| Dulaglutide | A10BJ05 | 0.16 | P | WHO ATC/DDD Index (A10BJ05) |
| Semaglutide | A10BJ06 | 0.11 | P | WHO ATC/DDD Index (A10BJ06) |
| Semaglutide | A10BJ06 | 10.5 | O | WHO ATC/DDD Index (A10BJ06) |
| Tirzepatide | A10BX16 | 1.4 | P | WHO ATC/DDD Index (A10BX16) |
